# Room-Temperature Phosphorescent Co-Crystal Showing Direct White Light and Photo-Electric Conversion

**DOI:** 10.3389/fchem.2021.765374

**Published:** 2021-11-04

**Authors:** Xiao-Gang Yang, Wen-Jing Qin, Ji-Rui Zhang, Xu-Ke Tian, Xin Fan, Lu-Fang Ma, Dongpeng Yan

**Affiliations:** ^1^ College of Chemistry and Chemical Engineering, Luoyang Normal University, Henan Province Function-oriented Porous Materials Key Laboratory, Luoyang, China; ^2^ College of Chemistry, Beijing Key Laboratory of Energy Conversion and Storage Materials, Beijing Normal University, Beijing, China

**Keywords:** room temperature phosphorescence, co-crystal, white light, triplet excitons, photoelectric response

## Abstract

The development of molecular crystalline materials with efficient room-temperature phosphorescence has been obtained much attention due to their fascinating photophysical properties and potential applications in the fields of data storage, bioimaging and photodynamic therapy. Herein, a new co-crystal complex [(DCPA) (AD)_2_] (DCPA = 9,10-di (4-carboxyphenyl)anthracene; AD = acridine) has been synthesized by a facile solvothermal process. Crystal structure analysis reveals that the co-crystal possesses orderly and alternant arrangement of DCPA donors and AD acceptors at molecular level. Fixed by strong hydrogen bonds, the DCPA molecule displays seriously twisty spatial conformation. Density functional theory (DFT) calculations show well separation of HOMO and LUMO for this co-crystal system, suggesting the efficient triplet excitons generation. Photoluminescence measurements show intensive cyan fluorescence (58.20 ns) and direct white phosphorescence (325 µs) emission at room-temperature. The transient current density–time curve reveals a typical switching electric response under the irradiation of simulated light, reveal that the [(DCPA) (AD)_2_] co-crystal has a high photoelectric response performance.

## Introduction

The rational design of molecular crystalline materials with long-lived room-temperature phosphorescence (RTP) has atracted tremendous attentions owing to their extended potential to create new opportunities in the development of photocatalytic reactions, photodynamic therapy, optical storage, organic light emitting diodes, and bioimaging (Bhattacharjee and Hirata, 2020; Jiang et al., 2018; Gao and Ma, 2021; Hirata, 2019; Gu et al., 2019; Lei et al., 2019; Wang et al., 2020; Wang et al., 2021; Li and Li, 2020; Yang et al., 2019; Yang et al., 2020; Gao et al., 2021; Chen et al., 2021). Besides the traditional noble-metal (ruthenium, platinum, iridium) based complexes (Xiang et al., 2013), breakthroughs have been achieved during the past decade on pure organics, polymers, metal–organic frameworks (MOFs), organic−inorganic hybrid perovskite and host–guest doping (Mu et al., 2017; Lu et al., 2019; Zhou and Yan, 2019; Lei et al., 2020; Liu et al., 2020; Lei et al., 2021; Wu et al., 2021; Zhou et al., 2021). Promising strategies (such as crystallization, H-aggregation, halogen bonding) have also been vastly accepted to obtain efficient RTP (Bolton et al., 2011; Gong et al., 2015; Kenry and Liu, 2019; Wang et al., 2020), and the inherent principle is absolutely focused on promoting triplet excitons generation.

Considering the spin-forbidden intersystem crossing (ISC) from excited singlet state to excited triplet state, the rate of ISC can be enhanced by reducing the energy gap (*ΔE*_ST_) between the lowest singlet excited state and a nearby triplet state. Small *ΔE*_ST_ can be achieved by designing the charge transfer of donor-acceptor system with large spatial separation between the HOMO and LUMO (Parke and Rivard, 2018). To date, many single component organic molecules with twisted donor-acceptor spatial conformation have been demonstrated as efficient RTP materials (Xiao et al., 2020; Xu et al., 2021). However, triplet state excitons of multi-component co-crystal donor-acceptor systems are still relatively limited (Zhou and Yan, 2019; Zhou et al., 2020), and the systematical investigation is needed for well understanding the relationship between their structures and photophysical behaviors.

In this paper, one new type of co-crystal [(DCPA) (AD)_2_] has been obtained under solvothermal conditions by the selection of 9,10-di (4-carboxyphenyl)anthracene (DCPA) electron donor and acridine (AD) electron acceptor. The obtained donor-acceptor co-crystal system shows alternant arrangement of DCPA and AD components at the molecular level. The crystal structure and density functional theory (DFT) calculations reveal that the DCPA molecule fixed by strong hydrogen bonds displays seriously twisty spatial conformation. This structure feature affords well separation of HOMO-LUMO, promoting for the generation of triplet excitons. As a result, the formation of [(DCPA) (AD)_2_] co-crystal exhibits cyan fluorescence and direct white long-lived RTP under ambient condition.

## Experimental

### Materials and General Methods

9,10-di (4-carboxyphenyl)anthracene (DCPA), acridine (AD) and anhydrous ethanol were purchased commercially. Single-crystal X-ray diffraction data were collected by Oxford Diffraction SuperNova area-detector diffractometer with the program of CrysAlisPro. The crystal structure was solved by SHELXS-2014 and SHELXL-2014 software (Sheldrick 2008). The crystallographic data for [(DCPA) (AD)_2_] were listed in Table 1. The CIF file (CCDC No. 2104581) presented in this study can be downloaded free of charge *via* http://www.ccdc.cam.ac.uk/conts/retrieving.html.

**TABLE 1:** Crystallographic data for [(DCPA) (AD)_2_].

Compound	[(DCPA) (AD)_2_]
Empirical formula	C_27_H_18_NO_2_
Formula weight	388.42
Crystal system	Triclinic
Space group	*P*ī
*a* (Å)	7.6078 (15)
*b* (Å)	9.2412 (15)
*c* (Å)	15.023 (2)
*α* (°)	94.670 (13)
*β* (°)	100.910 (15)
*γ* (°)	107.507 (16)
*V* (Å^3^)	978.2 (3)
*Z*	2
*D* (g cm^−3^)	1.319
*μ* (mm^−1^)	0.083
*R*_int_	0.0963
Goof	0.913
*R*_1_ (*I > 2σ* (*I*))	0.0907
*wR*_*2*_ (*I > 2σ*(*I*))	0.0913

Phase purity of co-crystal powders were tested by Bruker D8-ADVANCE X-ray diffractometer with Cu *Kα* radiation. Elemental analysis was performed by Perkin–Elmer Elementarvario elemental analysis instrument. Fourier transform infrared (FT-IR) spectra were measured by SHIMADZU IR Spirit-T spectrometer from 4,000 to 400 cm^−1^ with KBr pellet. UV-vis absorption spectra were detected by Shimadzu UV-3600 plus UV-vis-NIR spectrophotometer. Thermo gravimetric analysis (TGA) experiments were measured by SII EXSTAR6000 TG/DTA6300 thermal analyzer from room temperature to 800°C. The fluorescent and phosphorescent spectra were conducted on Edinburgh FLS1000 fluorescence spectrometer excited by xenon arc lamp (Xe900) and microsecond flash lamp, respectively. The time-resolved phosphorescent decay curves were measured by a microsecond flash lamp with a frequency of 100 Hz. Optoelectronic properties were tested on CHI 660 E electrochemical analyzer in a standard three-electrode system. The working electrode, counter electrode, reference electrode, and electrolyte is [(DCPA) (AD)_2_] powders modified indium tin oxide (ITO) glass, platinum wire, Ag/AgCl, and 0.5 M sodium sulfate aqueous solution, respectively. The linear sweep voltammetry (LSV) was recorded by the voltage rang of 0.2 to −1 V with a scan rate of 50 mV/s. The transient photocurrent were measured by on–off cycle’s illumination of Xe lamp (300 W) with bias potential (vs Ag/AgCl) of 0 and −0.5 V.

### Synthesis of [(DCPA) (AD)_2_].

A mixture of 9,10-di (4-carboxyphenyl)anthracene (0.1 mmol, 41.8 mg), acridine (0.2 mmol, 35.8 mg) and 8 ml EtOH was sealed into a Teflon reactor (23 ml), and heated at 120°C for 12 h. Then, the light yellow block crystals can be obtained after naturally cooled to room temperature. Anal. Calc (%) for C_27_H_18_NO_2_: C 83.48, H 4.67, N 3.61; found (%): C 83.12, H 4.33, N 3.46. IR (KBr pellet, cm^−1^): 3,415(w), 3,054(w), 1,947(w), 1,692(s), 1,607(m), 1,572(m), 1,524(m), 1,440(m), 1,401(m), 1,281(s), 1,100(m), 920(m), 853(w), 774(s), 735(s), 673(m), 505(m).

## Results and Discussion

### Crystal Structure Description

**FIGURE 1:**
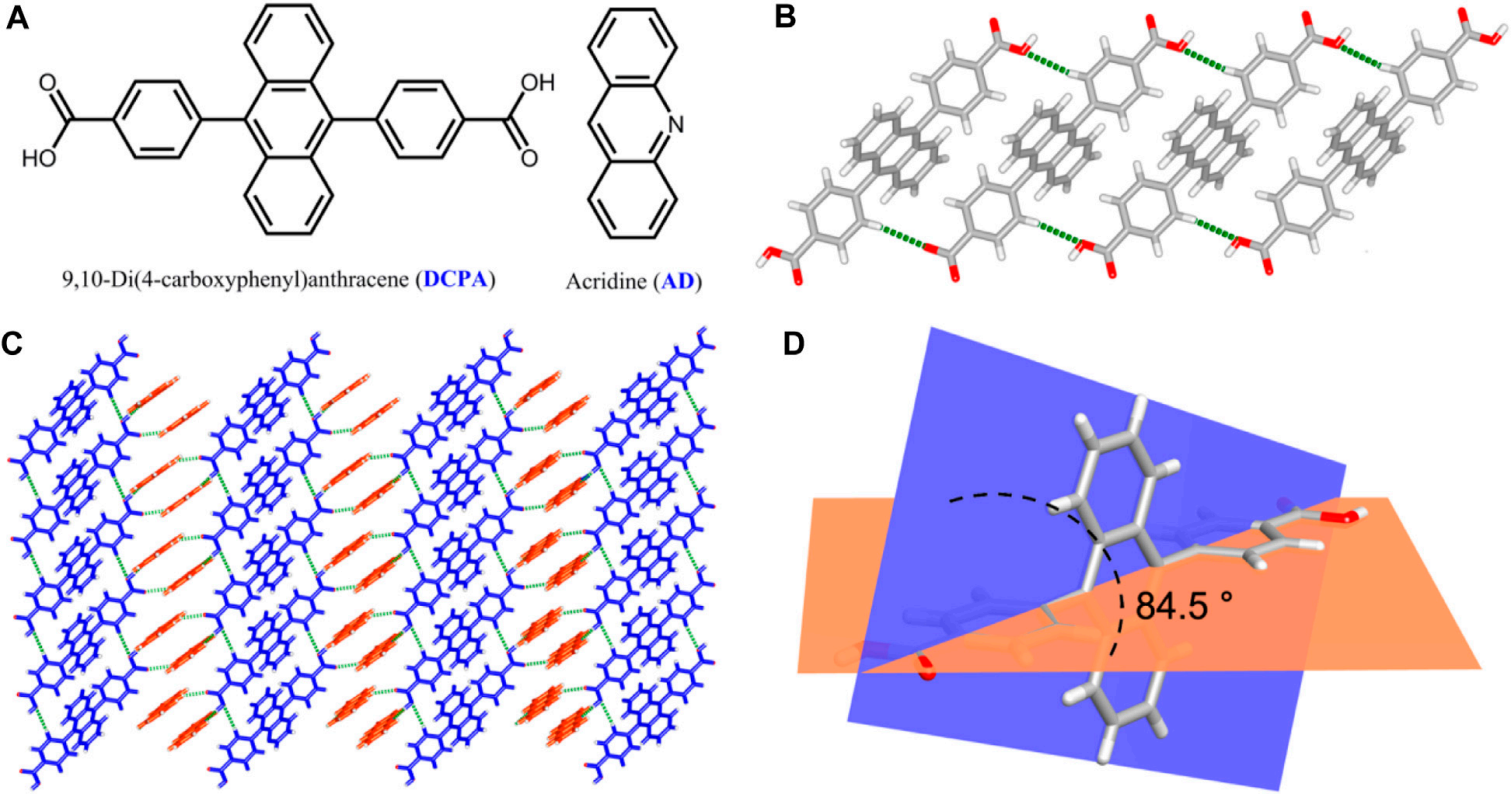
**(A)** Chemical structures of DCPA and AD molecules in this work. **(B)** 1D chain-like structure of DCPA extended by C‒H···O hydrogen bonds. **(C)** View of the 2D sheet constructed by the alternant arrangement of DCPA and AD molecules through C‒H···O and O‒H···N hydrogen bonds. **(D)** Torsion angles between benzene acid arm and the anthracene core.

High-grade light yellow block single crystals of the two-component [(DCPA) (AD)_2_] co-crystal were synthesized under the solvothermal condition from the mixture of DCPA and AD (Figure 1A) with a 1:2 stoichiometry in ethanol solution. Single-crystal X-ray diffraction analysis reveals that [(DCPA) (AD)_2_] crystallizes in triclinic *P*ī space group, and the asymmetric unit consists of two AD and one DCPA molecules. In the co-crystal system, the DCPA molecules are linked together by C‒H···O hydrogen bonds (C6‒H6···O2: H6···O2 = 2.69 Å, ∠C6‒H6···O2 = 141.30°) to form a 1D chain (Figure 1B). Pairs of AD molecules arrange in a head-to-tail *π*-stacking mode with short interplanar distance of 3.66 Å, which extends the DCPA 1D chain into a 2D sheet with the alternant arrangement of DCPA and AD molecules (Figure 1C).

Owing to above mentioned hydrogen bond interactions, the DCPA chromophores are highly fixed in an ordered arrangement at the molecular level, which exhibits a seriously twist conformation with torsion angles between benzene acid arm and the anthracene core up to 84.5° (Figure 1D). These supramolecular interactions also provide rigid environment to restrict the molecular motions/vibrations, minimizing the nonradiative loss of single/triplet excitons and facilitate for efficient emission (Yang et al., 2020).

### Powder X-ray Diffraction and Thermal Gravimetric Analysis

**FIGURE 2:**
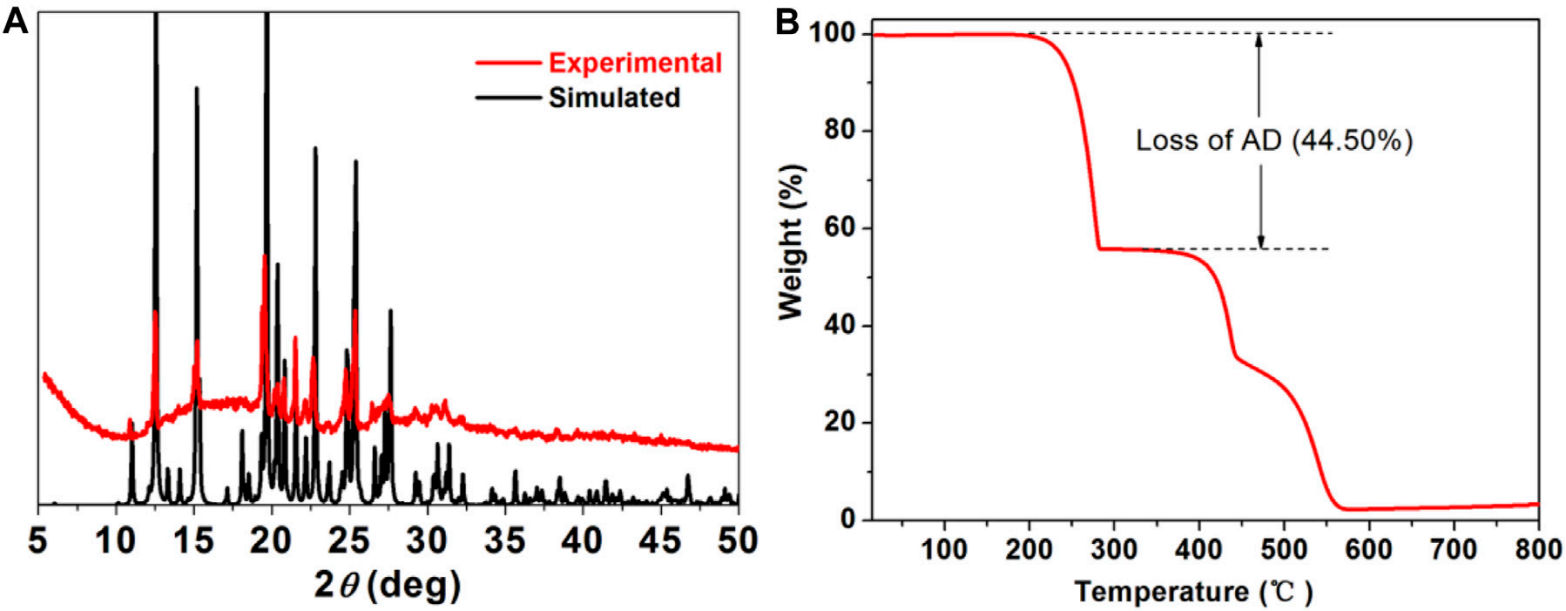
**(A)** PXRD patterns of simulated (black) and as synthesized **DCPA-AD** (red). **(B)** Thermo gravimetric analysis curve of **DCPA-AD**.

Powder X-ray diffraction (PXRD) experiment was conducted to detect the phase purity of [(DCPA) (AD)_2_] co-crystal (Figure 2A). The experiment diffraction peaks match well with the simulated one, providing the high purity and good crystalline degree of the as-synthesized samples. Thermo gravimetric (Figure 2B) curve shows the first weight loss of about 44.50% in the range of 200–283°C, assigning to the loss of AD molecules (calculated: 46.14%). Additional heating results in the gradual decomposition of framework of co-crystal.

## Photoluminescence Properties

**FIGURE 3:**
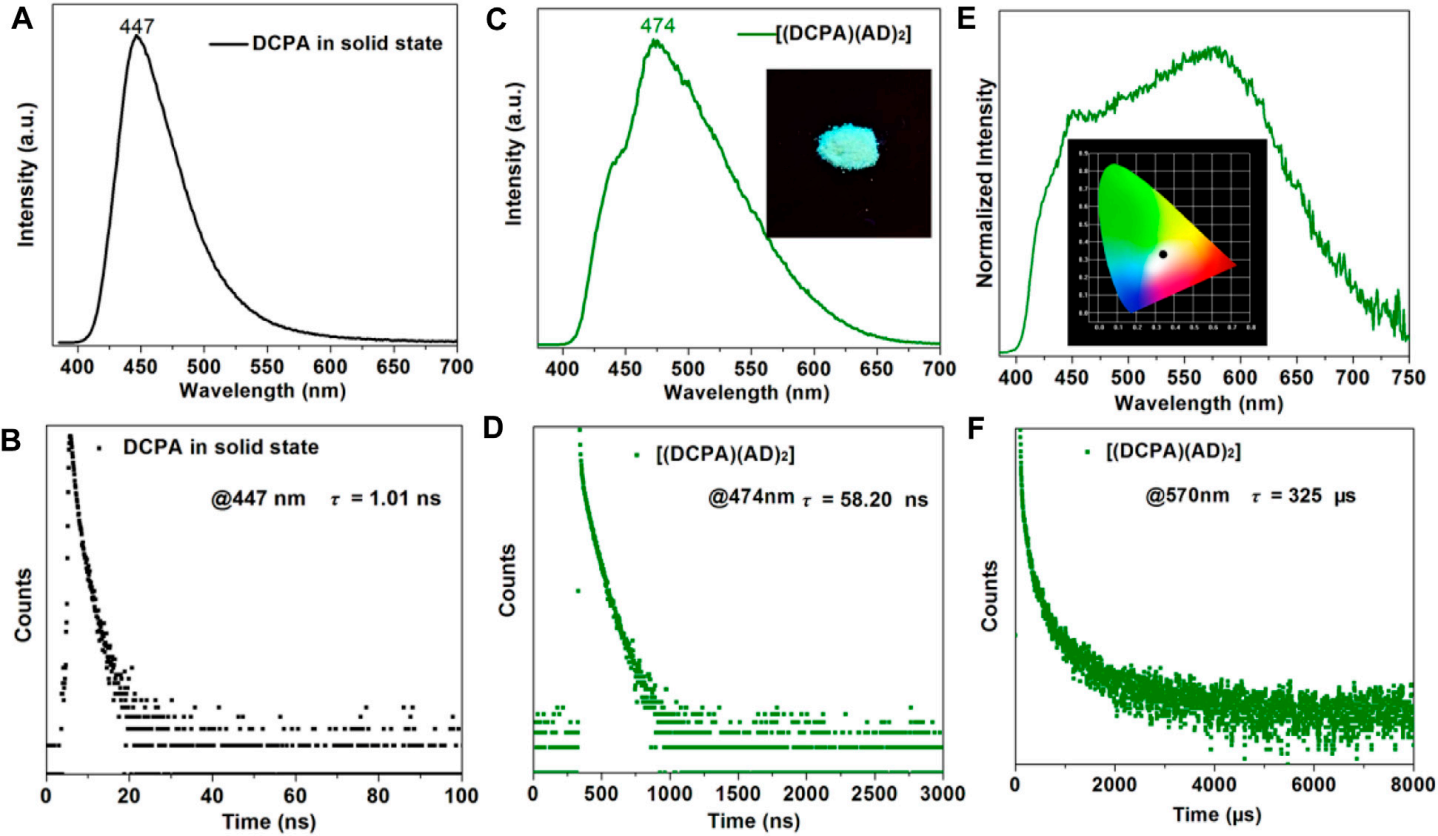
Fluorescence spectra **(A)** decay curve **(B)** of DCPA in solid state (λ_ex_ = 329 nm). Fluorescence spectra **(C)** decay curve **(D)** of [(DCPA) (AD)_2_] co-crystal in solid state (λ_ex_ = 365 nm). Insert shows solid state samples under UV (365 nm) light radiation. Phosphorescence spectra **(E)** decay curve **(F)** of [(DCPA) (AD)_2_] co-crystal in solid state (λ_ex_ = 365 nm). Insert shows CIE-1931 chromaticity diagram (0.33,0.34) of [(DCPA) (AD)_2_] co-crystal with white phosphorescence emission. All of these measurements were recorded under ambient condition.

The steady-state, transient-state photoluminescence (PL) spectra and time-resolved PL decay curves of both [(DCPA) (AD)_2_] co-crystal, pure DCPA and AD in solid state were recorded at room temperture. Figure 3A illustrates the fluorescence spectra of DCPA in solid state, which shows strong dark-blue emission owing to the presence of the anthracene chromophore (*λ*_ex_ = 329 nm). The fluorescence decay curve estimated at the maximal emission peak at 447 nm gives rise to a short lifetime of 1.01 ns(Figure 3B), whereas the single component AD has an emission peak at 396 nm and lifetime of 2.88 ns(Yang et al., 2020). By contrast, the formation of co-crystal presents a red-shift of the emission peak to long wavelength at 474 nm, attaching with a weak shoulder at about 443 nm when excited at 365 nm (Figure 3C), suggesting the charge transfer interaction between the DCPA donor and AD acceptor. Apart from the emission peak, the [(DCPA) (AD)_2_] co-crystal also shows much longer fluorescence lifetime up to 58.20 ns(Figure 3D), which is more than 50 times as long as that of free DCPA molecules in solid state.

The delayed PL spectrum shows a broad emission region spanning nearly the whole visible spectra with a maximum peak at 570 nm (Figure 3E). The time-resolved PL decay curve affords a long lifetime of 325 µs, indicating long-lived RTP emission of [(DCPA) (AD)_2_] co-crystal (Figure 3F). The inserts show the cyan emission of [(DCPA) (AD)_2_] crystalline powders irradiated under UV (365 nm) and the CIE-1931 chromaticity coordinate obtained from the phosphorescence spectra. The chromaticity coordinate of (0.33,0.34) is close to the optimum white-light with value of (0.33,0.33). The above results indicate that the fromation of co-crystal can largely tune the fluorescence emission of DCPA from dark-blue to cyan, and prolong the lifetime more than 50 times. In our opinion, the strong supramolecular interactions efficiently reduce the nonradiative loss of single/triplet excitons, and further enable prolonged PL lifetime.

### Density Functional Theory Calculations

**FIGURE 4:**
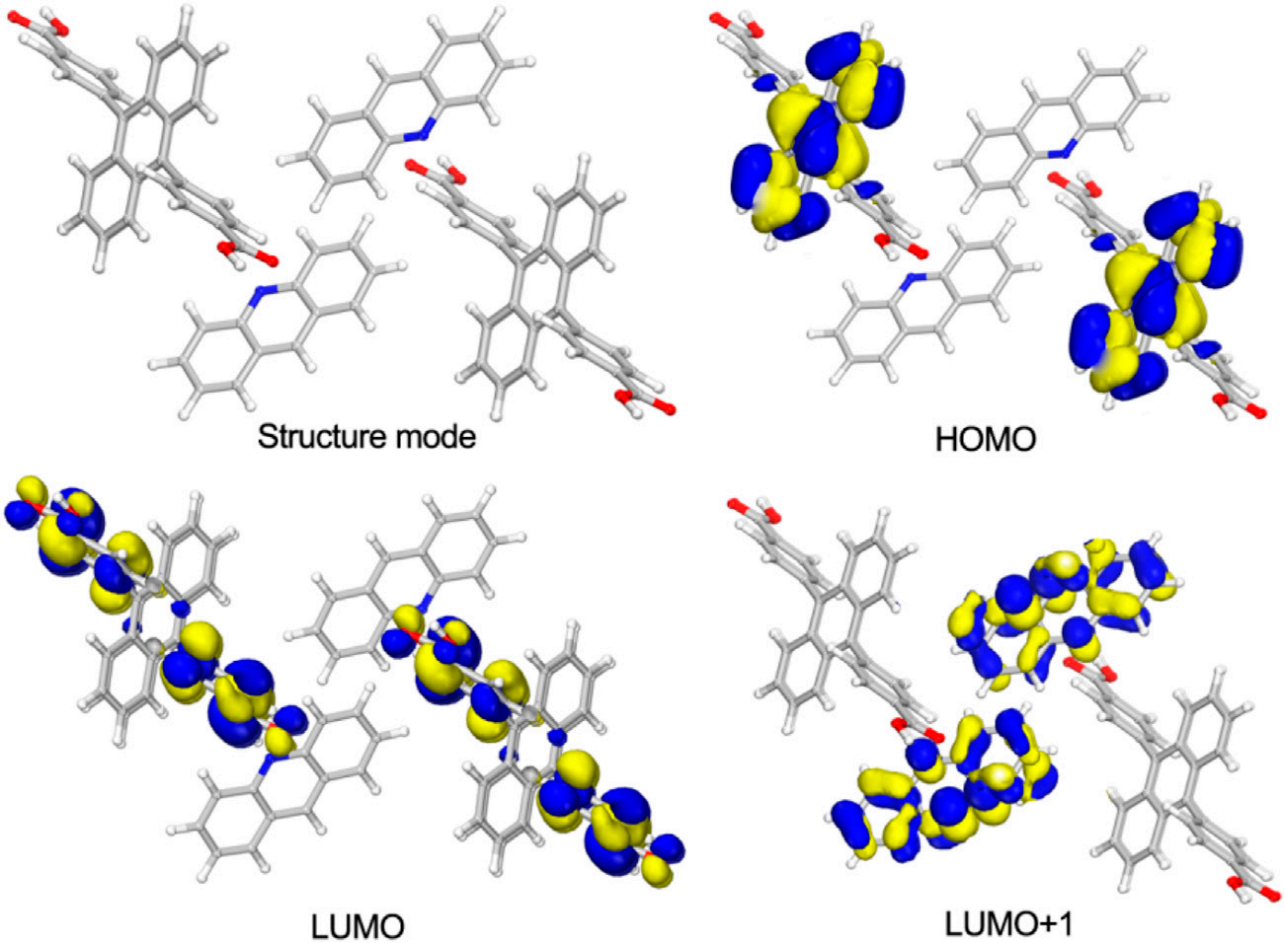
The structure mode and selected molecular orbitals of [(DCPA) (AD)_2_].

Density functional theory (DFT) calculations were conducted by Dmol^3^ module in Material Studio software package (Delley 2000) based on the X-ray single crystal diffraction data of [(DCPA) (AD)_2_]. The results show the highest occupied molecular orbital (HOMO) is occupied by the anthracene core of DCPA molecules, whereas the lowest unoccupied molecular orbital (LUMO) is exclusively located on the benzene acid groups. The LUMO+1 mainly appears on AD molecules (Figure 4). Herein, the seriously twist conformation of DCPA molecule leads to large spatial separation of the HOMO and LUMO. The alternant arrangement of DCPA electron donor and AD electron acceptor further promotes the separation of molecular orbitals, boosting the spin-orbit coupling and intersystem crossing for efficient triplet state exciton generation.

### Photo-Electronic Performance

**FIGURE 5:**
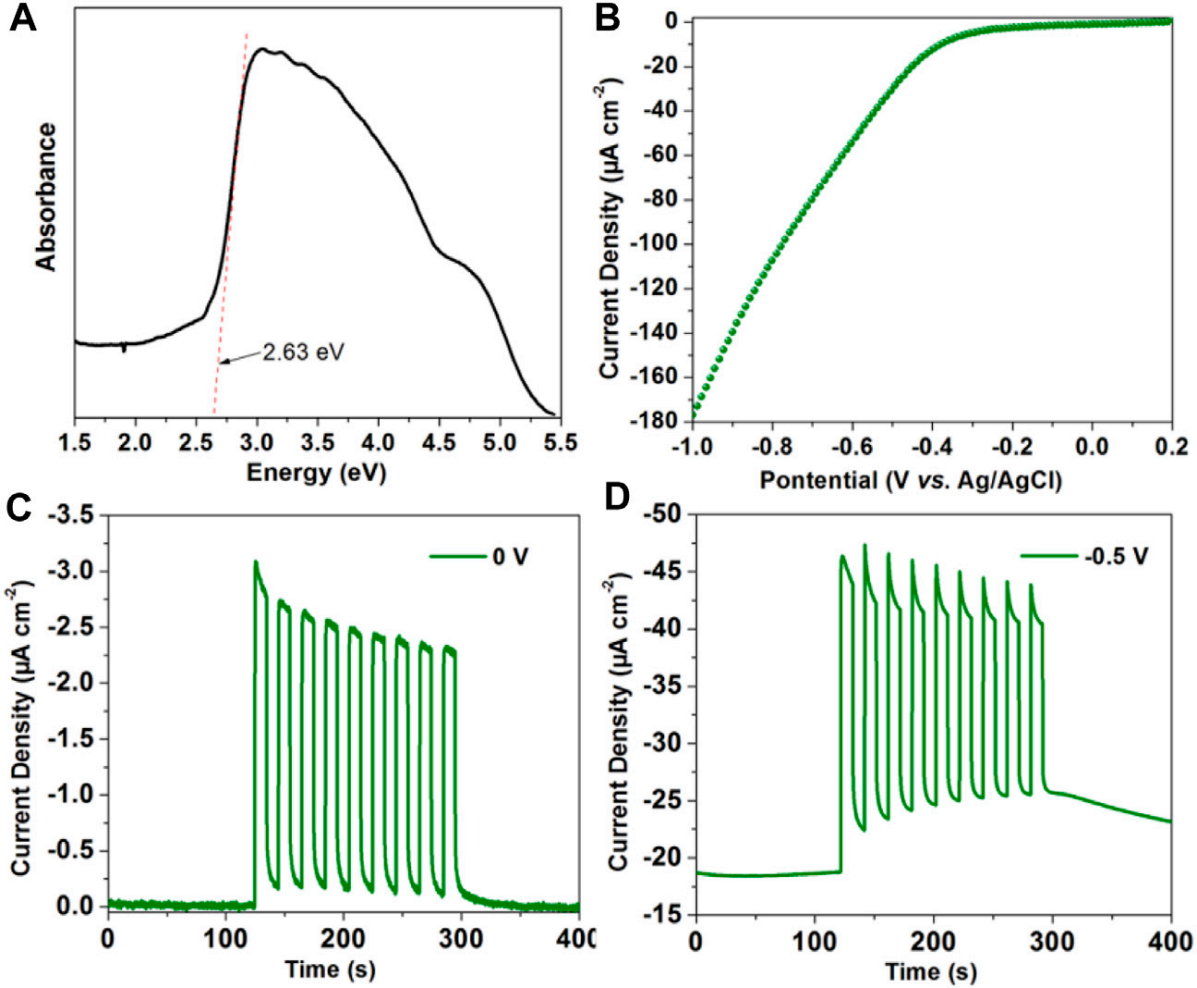
**(A)** UV-VIS-NIR absorption of as synthesized [(DCPA) (AD)_2_]. **(B)** The linear sweep voltammetry curve of as synthesized [(DCPA) (AD)_2_] modified ITO electrode measured in 0.5 M Na_2_SO_4_ aqueous solution. Transient current density–time curve of [(DCPA) (AD)_2_] at bias potential of 0 V **(C)** and −0.5 V **(D)** with the periodic on-off cycles of light radiation.

It has been found that the long-lived triplet state excitons have more chance for the electron migration (Yang et al., 2019). Encouraged by the long-lived RTP of [(DCPA) (AD)_2_] co-crystal in this work, its photo-electronic properties have been further conducted by a three-electrode system in Na_2_SO_4_ aqueous solution. The UV-Vis absorption spectrum shows an optical band gap of 2.63 eV (471 nm), consisting with the fluorescence emission peak (Figure 5A). The linear sweep voltammetry (LSV) curve reveals that the [(DCPA) (AD)_2_] co-crystal material can generate large current with the addition of negative potential (Figure 5B). The absence of redox peak suggests that the [(DCPA) (AD)_2_] co-crystal is stable within the applied bias potential from 0.2 to −1 V.

The transient current density–time curve reveals a typical on/off switching response under the irradiation of simulated light. Without only bias potential, it generates high photocurrent up to −3.1 μA cm^−2^ with the momentary light radiation. Under the initiatory dark condition, extremely small dark current of about 0.002 μA cm^−2^ can be detected (Figure 5C). The rate of current between light radiation and dark conditions was calculated up to 1,550. By the addition of −0.5 V bias potential, it generates more large current of about −46.5 μA cm^−2^ under light radiation (Figure 5D). All these results reveal that the [(DCPA) (AD)_2_] co-crystal has superior photoelectric response performance, which can be applied in the future photoelectric detector device.

## Conclusion

In summary, we report a rare example of direct white-light RTP co-crystal material [(DCPA) (AD)_2_], which can be synthesized under a facile solvothermal condition. The framework of [(DCPA) (AD)_2_] shows an orderly distribution of heterojuction at the molecular level: alternant arrangement of DCPA electronic donors and AD electron acceptors bonded together through strong C‒H···O and O‒H···N hydrogen bonds. Fixed by these supramolecular interactions, the molecular motions/vibrations can be restricted, which affords long-lasting singlet and triplet excitons through minimize the nonradiative loss. In addition, the seriously twist conformation of DCPA molecule is beneficial to the separation of molecule orbitals. Combined with the introduction of AD acceptor, it provides efficient platform for long distance exciton transfer and good electron-hole separation ability, possessing superior photoelectric response performance. Therefore, this work not only develops a new type of white-light RTP co-crystal, but also provides a perspective to deeply understand the relationship among molecular structure, stacking mode and photoelectric performance.

## Data Availability

The data presented in the study are deposited in the (Cambridge Crystallographic Data Centre) repository, accession number (2104581).
